# Genetic Neonatal-Onset Epilepsies and Developmental/Epileptic Encephalopathies with Movement Disorders: A Systematic Review

**DOI:** 10.3390/ijms22084202

**Published:** 2021-04-18

**Authors:** Carlotta Spagnoli, Carlo Fusco, Antonio Percesepe, Vincenzo Leuzzi, Francesco Pisani

**Affiliations:** 1 Child Neurology Unit, AUSL-IRCCS di Reggio Emilia, 42122 Reggio Emilia, Italy; carlo.fusco@ausl.re.it; 2 Medical Genetics, Department of Clinical and Experimental Medicine, University Hospital of Parma, 43126 Parma, Italy; antonio.percesepe@unipr.it; 3 Department of Paediatrics, Child Neurology and Psychiatry, Sapienza University of Rome, Via dei Sabelli 108, 00185 Rome, Italy; vincenzo.leuzzi@uniroma1.it; 4 Child Neuropsychiatry Unit, University Hospital of Parma, 43126 Parma, Italy; francesco.pisani@unipr.it

**Keywords:** newborn, epilepsy, epileptic encephalopathy, developmental encephalopathy, movement disorder, monogenic

## Abstract

Despite expanding next generation sequencing technologies and increasing clinical interest into complex neurologic phenotypes associating epilepsies and developmental/epileptic encephalopathies (DE/EE) with movement disorders (MD), these monogenic conditions have been less extensively investigated in the neonatal period compared to infancy. We reviewed the medical literature in the study period 2000–2020 to report on monogenic conditions characterized by neonatal onset epilepsy and/or DE/EE and development of an MD, and described their electroclinical, genetic and neuroimaging spectra. In accordance with a PRISMA statement, we created a data collection sheet and a protocol specifying inclusion and exclusion criteria. A total of 28 different genes (from 49 papers) leading to neonatal-onset DE/EE with multiple seizure types, mainly featuring tonic and myoclonic, but also focal motor seizures and a hyperkinetic MD in 89% of conditions, with neonatal onset in 22%, were identified. Neonatal seizure semiology, or MD age of onset, were not always available. The rate of hypokinetic MD was low, and was described from the neonatal period only, with WW domain containing oxidoreductase (*WWOX)* pathogenic variants. The outcome is characterized by high rates of associated neurodevelopmental disorders and microcephaly. Brain MRI findings are either normal or nonspecific in most conditions, but serial imaging can be necessary in order to detect progressive abnormalities. We found high genetic heterogeneity and low numbers of described patients. Neurological phenotypes are complex, reflecting the involvement of genes necessary for early brain development. Future studies should focus on accurate neonatal epileptic phenotyping, and detailed description of semiology and time-course, of the associated MD, especially for the rarest conditions.

## 1. Introduction

Neonatal-onset epilepsies follow a different time course and evolution from acquired symptomatic neonatal seizures [1]. Acute symptomatic neonatal seizures are sustained by acute, acquired injury to the central nervous system, notably hypoxic-ischemic encephalopathy, intraventricular hemorrhage, perinatal ischemic stroke or, less frequently, infections or transient metabolic derangement. They typically tend to cease spontaneously after the first days of life, but can be followed by the occurrence of recurrent spontaneous seizures (epilepsy) later in life in a substantial proportion of patients. On the contrary, neonatal seizures caused by structural (genetically driven) or nonstructural inborn metabolic or genetic etiologies are self-sustained phenomena, which must be considered as early onset epilepsies [2].

With the advent of broad sequencing methods, improved diagnostic yield has been reached, and these are now estimated to represent 10%–15% of all neonatal seizures [3].

In the last few years, our awareness of the association between genetically determined epilepsies, or developmental/epileptic encephalopathies (DE/EE) with movement disorders (MD), has increased; in some cases, delineating specific phenotypes [4,5,6]. While this association in the neonatal period is most frequently caused by neurometabolic disorders [7], nonmetabolic monogenic conditions also have to be taken into account. However, the occurrence of both these features during the neonatal period is still under investigated, and literature data are limited.

Therefore, we undertook a systematic literature review to describe the phenotypic and genotypic spectra of MD in genetic neonatal-onset epilepsies and DE/EE.

## 2. Methods

### 2.1. Protocol

A systematic literature review, according to a PRISMA statement [8], was performed. A protocol specifying inclusion/exclusion criteria, outcome measures, and analytical methods was created.

#### 2.1.1. Eligibility Criteria

Inclusion criteria: studies conducted on humans focusing on genetic epilepsies and/or DE/EE (as defined by ILAE [9]) with neonatal-onset cases with onset of MD(s), as classified in [10] at any age; proven monogenic etiology, demonstrated by detecting definitely or likely pathogenic variants according to ACMG criteria [11] (by single gene testing, targeted next-generation sequencing panels or whole exome sequencing); written in English; published between 1 January 2000 and 31 December 2020. Ages at MD onset were classified as: neonatal/early infantile (birth–4 months), infantile (5–12 months), toddler (1–3 years), early childhood (3–8 years), late childhood (9–11 years), adolescence (12–18 years), and adulthood (≥18 years).

Exclusion criteria: Reviews, authors’ replies and commentaries, book chapters, non-English language papers, animal experiments, articles on structural, autoimmune or neurometabolic etiologies; molecular diagnosis unavailable.

#### 2.1.2. Information Sources

PubMed and Scopus databases. Reference lists were reviewed in order to collect additional papers.

#### 2.1.3. Search

Our search terms were: (neonatal) AND (epileptic encephalopathy) OR (epilepsy) AND (movement disorder).

#### 2.1.4. Study Selection

Results were reviewed by title, abstract and by full-text review.

#### 2.1.5. Data Items

Data collection sheet is available as supplementary material.

The quality of evidence was rated according to [12] (Table S1).

## 3. Results

**Figure 1:**
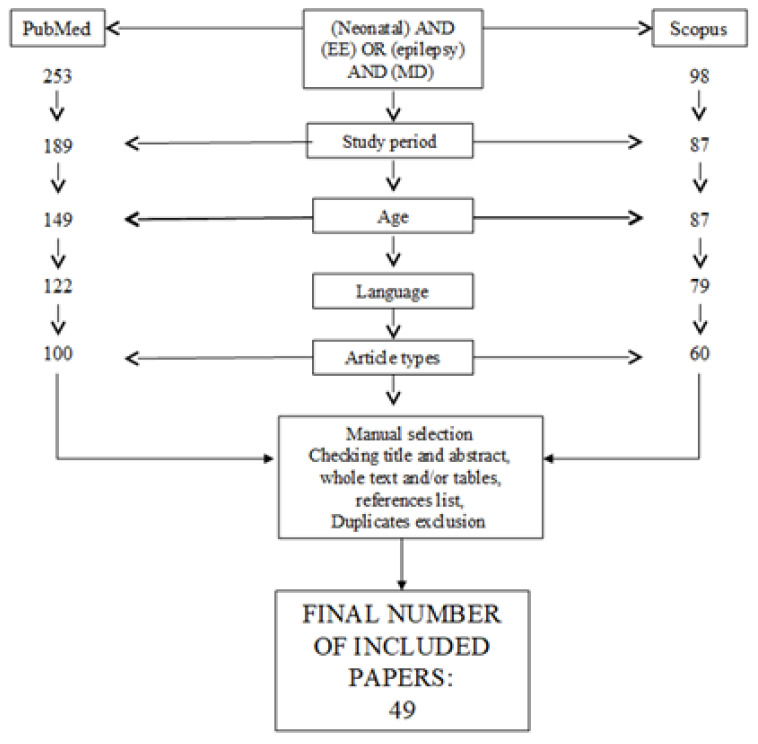
Articles selection flow-chart.

Articles’ selection flow-chart is detailed in Figure 1.

A total of 49 studies on 27 genes satisfied the inclusion criteria. We identified more than one paper for eight genes: *ATP1A3* (one case series [13], one case report [14]); *GNAO1* (four original articles [15,16,17,18], one case report [19]); *KCNQ2* (two original articles [20,21], one case series [22], two case reports [23,24]); *SCN8A* (three original articles [6,25,26], two case reports [27,28]); *SLC13A5* (two original articles [29,30]); *STXBP1* (two original articles [31,32], one case series [33], one case report [34]); *SCN2A* (three original articles [35,36,37] and two case reports [38,39]); and *SYNJ1* (one original article [40] and one case report [41]).

### 3.1. Genetic Results

The majority of analyzed conditions are autosomal dominant (AD), 5 five (*AP3B2*, *PCDH12*, *SLC13A5*, *SYNJ1* and *WWOX*) are autosomal recessive, and two are X-linked (*CDKL5* and *SMC1A*).

Segregation analysis was performed in all cases except: 2/33 patients within the *CACNA1E* cohort (parents unavailable) [42], and 2/17 cases with *SCN2A* variants (father unavailable, mother tested negative) [36].

Among AD conditions, detected variants were de novo in all cases except the 2 related patients with the *FHF1* variant (suspected germline mosaicism) [43], 1/7 cases with *GNAO1* variants (suspected germline mosaicism) [15], 2/17 unrelated cases with paternally inherited *SCN2A* variants [36,37], and 1 *KCNQ2* variant, inherited from the affected mother [22].

In tested AR conditions, parents were heterozygous carriers [29,30,44,45,46].

For X-linked conditions [47,48], variants were confirmed to be de novo (Table S1).

Classification according to gene function is reported in Table 1.

### 3.2. Clinical Findings

#### 3.2.1. Epilepsy

The main seizure types at onset include “focal” (30 patients, 14 with clonic seizures), tonic (24), epileptic spasms (ES) (14), and myoclonic (10) [6,15,17,18,19,22,25,26,27,28,31,32,33,34,35,39,42,43,44,45,46,47,48,49,50,51,52,53,54,55,56]. Thirteen patients had tonic-clonic seizures [6,15,16,26,33,54,56,57], twelve had subtle seizures [19,29,30,36,38,39,41,44,46,54,58], two had “multifocal” seizures [36,52] and one had “generalized” [57]. No information on neonatal seizure semiology is available in three [13,14,59] (Table S1). A high rate of status epilepticus is reported with *SLC13A5* pathogenic variants [29,30]. KCNQ2-related encephalopathy is associated with focal tonic, followed by clonic jerks and frequent dysautonomic features. Similar semiology of tonic and/or clonic phenomena, possibly with deaturation or apnea, can also be detected in SCN2A-positive patients.

The most frequent electroclinical diagnosis is early onset EE (EOEE), reported for 22/27 (81.5%) genes. Electroclinical classification according to genetic diagnosis is detailed in Table 2.

#### 3.2.2. Neurological Examination

Hypotonia (especially axial) can be present in 22 (81.5%) conditions: *AP3B2*, *ATP1A3*, *CACNA1E*, *CDKL5*, *DNM1*, *FHF1*, *GABRB2*, *GABRB3*, *GABRG2*, *GRIA2*, *GNAO1*, *KCNQ2*, *PCDH12*, *PURA*, *RHOBTB2*, *SCN2A*, *SCN8A*, *SLC13A5*, *STXBP1*, *SYNJ1*, *VAMP2*, and *WWOX;* microcephaly in 12 (44%): *AP3B2*, *DNM1*, *FHF1*, *GABRA1*, *GABRB2*, *GRIA2*, *GNAO1*, *PCDH12*, *SCN8A*, and *SLC13A5* (absent in neonatal-onset cases), postnatal in *ATP1A3*, head growth deceleration in *CDKL5.* On the contrary, macrocephaly is rare (*CACNA1E*) [42].

#### 3.2.3. Neurodevelopmental Aspects

DD and/or intellectual disability (ID) can develop in all disorders, while autistic features are described in 6/27 (22%: *CDKL5*, *KCNQ2*, *GRIN2B*, *SCN2A*, *STXBP1*, and *VAMP2*), regression in 7/27 (26%: *AP3B2*, *CACNA1E*, *CDKL5*, *FHF1*, *GRIN2B*, *SCN8A*, and *STXBP1*), and stagnation in 2 (7%: *GABRB3*, *GNAO1*).

#### 3.2.4. Movement Disorder

MD is hyperkinetic in 23/27 (85%), while a hypokinetic disorder can develop in *WWOX-*, *STXBP1-*, and *GRIA2-*related disorders (Table 3; Table S1).

Age of MD onset was reported for 10/27 (37%) monogenic conditions. Neonatal onset is described in 6 (myoclonus in *GRIA2*, *KCNQ2*, *SCN8A* and *STXBP1*, tremor in *SCN8A*, dystonia in *PCDH12*, non-epileptic tonic and dystonic events, and episodic oculomotor abnormalities with tachycardia in *ATP1A3*). Infantile onset is described for paroxysmal non-epileptic events and athetosis in *ATP1A3*, excessive startle, focal dystonia and reduced spontaneous movements in *GNAO1*, and dystonia and opistotonic posturing in *SYNJ1.* Between 1 and 3 years, hypokinesia and oculogyric crises develop in *GRIA2*, and stereotypies in *SMC1A* and *STXBP1*, while episodic ataxia develops in *SCN2A*-positive patients with previous benign familial infantile seizures (BFIS). In patients harboring *STXBP1* pathogenic variants, ataxia and head nodding are reported from early childhood, while dystonia and tremor in late childhood. Parkinsonism can take place from adolescence. Non-further specified “early onset” dystonia, chorea, dyskinesia, myoclonus and non-specified hyperkinetic MD are reported with *CACNA1E*, whereas, in the remaining conditions, age of MD onset was not reported.

### 3.3. Neuroimaging Findings

Brain MRI is unremarkable in most disorders (Table 4). Nonspecific findings include cerebral atrophy in 10 (10/27, 37%), white matter abnormalities (including hypomyelination) in 7 (7/27, 26%), and myelination delay in 7 (7/27, 26%). The range of additional findings is wide but shared by fewer conditions. *PCDH12*-related disorder has specific findings of midbrain, hypothalamus and optic trait dysplasia (Table 4).

### 3.4. Presentation and Outcome According to Genetic Diagnosis

#### 3.4.1. Enzymes

*WWOX *(WW domain-containing oxidoreductase): 5/20 cases had neonatal-onset epilepsy with associated MD. One additional patient also had suspected paroxysmal non-epileptic events. Seizure types at onset are described as focal clonic, focal non-specified, occipital, subtle (one each), and ES in two. Onset ranged between days 1 and 20. During follow-up, seizure types included spasm, tonic, tonic-clonic and absences. EEG can present interictal focal, or multifocal, discharges with posterior predominance. Associated findings include hypotonia with pyramidal signs and/or appendicular hypertonia, early onset scoliosis and/or kyphosis and frequently poor/absent eye contact, in some cases with abnormal evoked potentials or electroretinogram, and possible retinal degeneration. MD is strikingly hypokinetic with very limited spontaneous movements, while upper limbs’ dystonia, myoclonus, startles, paroxysmal involuntary movements, and episodes of boxing/pedaling were also described [46].

#### 3.4.2. Synaptopathies

*AP3B2* (adaptor-related protein complex 3, beta-2 subunit): EOEE characterized by severe/profound DD, with possible psychomotor deterioration, poor visual contact with optic atrophy, and postnatal microcephaly. Patients usually present in infancy, but 2/12 (16.7%) cases had neonatal onset with hypertonia and seizures (subtle in one and tonic in the second). EEG data are unavailable. Neurodevelopmental outcome is characterized by severe-to-profound DD and midline stereotypies. Additional phenotypes include dyskinesias and choreoathetosis. Age at onset and subsequent evolution are not reported. Epilepsy is usually drug-resistant, but in one of the two neonatal-onset cases, seizure freedom is reported [44].

*DNM1 *(dynamin 1): The common phenotype includes severe-to-profound ID, hypotonia and frequent (76%) EE characterized by infantile spasms, frequently evolving into Lennox–Gastaut syndrome and drug resistance. The mean age at epilepsy onset is 7.6 months. However, 1/21 patients had neonatal-onset myoclonic epilepsy at 3 weeks of age, later followed by absence seizures in the context of profound DD, hypotonia followed by spasticity, microcephaly and dystonia. EEG shows background slowing. Long-term epilepsy outcome is reportedly good [49].

*STXBP1* (syntaxin-binding protein 1): In two case series, 2/5 [33] and 4/5 [31] had neonatal-onset DEE with MD. Reported seizure semiology at onset includes mainly tonic, myoclonic and ES, although clonic and tonic-clonic seizures were also described. Seizure types at follow-up, when reported, include persistence of ES and tonic seizures, although focal impaired awareness, gelastic, and tonic-clonic are also enumerated. EEG is abnormal (either burst-suppression or multifocal). One case series reports seizure freedom from 6 months of age in 2 patients, and at 12 and 18 months in one each. Hypotonia, severe DD, and autistic features are frequent. The associated MD can be complex. Myoclonus has been described since the neonatal period, while stereotypies after 2 years, ataxia in childhood, and, interestingly, parkinsonian features were reported in two individuals (in one from 12 years, and in the second—followed up until 45 years of age—with unspecified onset). Head nodding, dystonia, chorea or choreathethosis, and dyskinesias are additional features with an unspecified age-of-onset [31,34].

*VAMP2 *(vesicle-associated membrane protein 2): 2/5 patients had hyperkinetic MD and epilepsy, with neonatal-onset in one. Seizure semiology is reported as initially focal, with ictal EEG characterized by fast rhythmic activity, evolving into sharp-and-slow-wave complexes, and, later, tonic-clonic seizures. The neurological phenotype is significant for hypotonia, cerebral visual impairment, severe ID with autistic and Rett-like features, and self-injurious behaviour. The patient also developed generalized chorea of unspecified onset [56].

SYNJ1 (Synaptojanin 1): Two articles report on three patients affected by neonatal-onset refractory DEE, an early onset neurodegenerative course and premature death (between 2 years, 4months and 8 years), caused by biallelic variants in the *SYNJ1* gene. Seizure onset occurred between D1 and D12 of life, with eye blinking, hypertonus, bicycling, or eye deviation. EEG was abnormal/severely abnormal with multifocal discharges in all. Neurological examination progressed from hypotonia to severe spastic quadriparesis and dystonia with/without opistotonic posturing, developing since infancy (with partial response to clonazepam in one). DD is profound and accompanied by cortical visual impairment (2/3). In one case, thin corpus callosum, atrophy and gliosis were detected on a brain MRI, but the remaining two patients had normal findings when imaged [40,41].

#### 3.4.3. ATPASEs

*ATP1A3* (sodium–potassium ATPase, alpha3 polypeptide): EOEE with seizure onset at 4 h and on the second day of life in two term newborns is described. No further description is provided. Subsequent refractory epilepsy was reported, but ictal semiology is not described. Both patients developed postnatal microcephaly and severe DD. Non-epileptic episodes featuring nystagmus, dysconjugate gaze, and decreased responsiveness started at 2 months of age, and athetotic movements from 11 months in one patient. The second experienced non-epileptic tonic and dystonic events, and episodic oculomotor abnormalities with tachycardia since the neonatal age [13,14].

#### 3.4.4. Channelopathies

*CACNA1E* (calcium channel, voltage dependent, alpha-1E subunit): DE/EE with contractures, macrocephaly, and dyskinesias is reported. A total 4/30 (13%) cases experienced neonatal onset of seizures: two with myoclonic seizures and two with ES. None developed an MD, in contrast with the rest of the cohort, in which dystonia, dyskinesias, chorea and myoclonus were reported [42].

*KCNQ2 *(potassium channel, voltage gated, KQT-like subfamily, member 2): Typical seizure semiology consists of tonic versive seizure with clonic component and possible associated autonomic features (i.e., apnea). Neonatal EEG is severely abnormal, with multifocal random suppression with multifocal discharges. An association with hyperkinetic MD has been increasingly documented: dystonia or spastic-dystonic tetraparesis [20,21,23], paroxysmal “myoclonus-like” dyskinesia (reported in mother and son), ataxic/broad-based gait (two patients), hyperkinetic dyskinesia, mild distal tremor (at 2 years of age), and hand stereotypies (three reports). There is a single report on the occurrence of fever-sensitive chorea and myoclonus [24].

*KCNMA1* (potassium channel, calcium-activated, large conductance, subfamily M, alpha member 1): *KCNMA1* pathogenic variants were first reported in a family with AD generalized epilepsy and paroxysmal non-kinesigenic dyskinesia. Typical age of onset is in infancy. In one paper, non-specified neonatal-onset seizures were reported in the context of severe DD, in association with later development of paroxysmal non-kinesigenic dyskinesia [59].

*SCN2A* (sodium channel, voltage-gated, type II, alpha subunit): 17 patients from 5 papers experience neonatal-onset epilepsy and subsequent MD. Of note, 6 harbor the same c.788C > T missense variant, which is a known hot spot, while 7 patients have a neonatal-onset DE/EE [34], and 10 have a benign-familial-infantile (BFIS) type 3 (BFIS3) phenotype. Seizures in the neonatal period are reported as focal, with multiple seizure types in all patients with DEE, including tonic and spasms; also, patients with a milder phenotype tend to present with tonic seizures, with/without a (hemi)clonic component and autonomic changes (apnea and desaturations). In one case, hypomotor semiology is described at seizure onset. In patients with DEE, EEG is normal or slow in five, and burst-suppression in two, but all with multifocal discharges, while mildly abnormal/normal background with focal sharp has been reported in BFIS3-cases. No cases experienced regression. Neurological examination is characterized by axial hypotonia in nine and appendicular hypertonia in four. DD is severe/profound in five, mild/moderate in three and normal in three. Autistic features are reported in just one case, in keeping with early onset epilepsy cases resulting from gain-of-function variants. MD is either in the form of stereotypies, opisthotonus or chorea (1 patient), oculogyric crises (2 patients), and dystonia (4 patients) [35], or in the form of episodic ataxia (10 cases, following BFIS3). Seizure control is reported in seven patients (between 3 and 13 months of age, when available), while episodic ataxia is ongoing, with variable response to acetazolamide. Brain MRI findings have been reported in one paper, showing white matter changes in seven cases, basal ganglia involvement in six, and brainstem involvement in two [36], while neuroimaging data in BFIS3 followed by episodic ataxia were not available [36,37,38,39].

*SCN8A* (sodium channel, voltage-gated, type VIII, alpha subunit): Neonatal-onset epilepsy with associated MD was reported in 1/22 [6], 1/19 [25] and 2/17 [26] cases, respectively. Seizure types are described as tonic with/without apnea, spasms, but also focal clonic. Multiple seizures develop during follow-up (tonic-clonic, focal with impaired awareness, episodes of status epilepticus). EEG, when reported, is slow, low voltage, or “burst-suppression-like” during sleep. Deterioration has been highlighted, and a posterior (temporo-parieto-occipital) prevalence of interictal discharges is consistently found [6]. Hypotonia, acquired microcephaly, and severe/profound DD are constant. Poor eye contact and cortical blindness seem very common (17/22 cases from the whole cohort in [6]). Coarse tremor, non-epileptic myoclonus, and exaggerated startle have been reported at birth, while dyskinesia, paroxysmal dystonic posturing, ataxia and dystonia have also been described, with unspecified age of onset [6].

#### 3.4.5. Transcription Regulators

*CDKL5* (cyclin-dependent kinase-like 5): Out of seven diagnosed cases, two had neonatal-onset epilepsy, with myoclonic and focal seizures. Neonatal EEG is not reported, but subsequent recordings show slow background with intermittent multifocal discharges. Both developed hand stereotypies with lack of purposeful hand movements, with dysautonomic features in one. Poor eye contact is present in both, and overt autistic features in one. In the whole cohort, choreiform movements are also described [47].

*PURA* (purine-rich element-binding protein A): A single patient out of 30 has been reported with neonatal-onset myoclonic seizures and moderate ID. Hand stereotypies and exaggerated startle response are described as consistent, but their presence is unknown in the only patient with neonatal-onset epilepsy. Other patients with PURA syndrome develop dystonia or ataxia/broad-based gait [55].

*SMC1A *(structural maintenance of chromosomes 1A): One patient with focal epilepsy, beginning within the first month, of life has been described. Semiology in the neonatal period was characterized by eyelid myoclonia, evolving into drug-resistant multifocal epilepsy. The patient was born with IUGR and congenital microcephaly. She developed hand stereotypies at 2 years of age. Outcome is significant for severe ID, spastic tetraparesis, poor eye contact. Additional clinical features include reflux, scoliosis, and dysmorphisms. *SMC1A* pathogenic variants have been associated with Cornelia de Lange syndrome, but, at both extremes of the spectrum, severe cases with early onset epilepsy/EE or mildly affected patients are described [48].

#### 3.4.6. Transcription Factors

*FOXG1* (forkhead box G1): *FOXG1* pathogenic variants are associated with a complex neurodevelopmental phenotype (postnatal microcephaly, DD and hyperkinetic MD with prominent stereotypes, and epilepsy), with a mean onset in early childhood. Some genotype-phenotype correlations have emerged, as frameshift and nonsense N-terminus mutations are associated with greater severity and MRI anomalies. One case of neonatal-onset epilepsy is reported in a series of 45 patients. The patient, now aged 8 years, is still experiencing less than one focal seizure/month [50].

#### 3.4.7. Growth Factors

*FGF12 (FHF1)* (fibroblast growth factor 12): Two siblings affected by neonatal-onset EE with tonic seizures and a characteristic neonatal EEG pattern (interictal: severe background slowing and multifocal discharges, ictal: low voltage fast activity, followed by prolonged background suppression) with normal neurologic status prior to seizure onset, subsequently developed a complex phenotype with hypotonia, microcephaly, feeding difficulties, cerebral visual impairment with pale optic nerve disc, and limb ataxia. Both developed cerebellar atrophy in their childhood. Follow-up EEG reported in the first sibling at 5 months was hypsarrhythmic, with no detected infantile spasms. Disease course was degenerative and both died before age 7 [43].

#### 3.4.8. Receptors

*GABRA1* (gamma-aminobutyric acid receptor, alpha1): Although mean age at epilepsy onset is 2.6 months, in a series of 6 EOEE patients [51], two had neonatal onset EE (tonic in one and myoclonic—evolving to infantile spasms at 1.5 months—in the second). Clinical evolution of both patients is characterized by severe DD with intractable epilepsy in one, and progressive brain atrophy. The complex neurologic phenotype also features non-epileptic myoclonus in one and choreoathethosis (resolving at 2 years of age) in the second [51].

*GABRB2* (gamma-aminobutyric acid receptor, beta-2): One neonatal-onset case is found out of seven (1/7, 14%). Epileptic seizures were described as tonic, focal and multifocal. EEG background is abnormal with multifocal discharges. Subsequent development is characterized by severe DD with hypotonia (followed by spasticity) and dystonia, and acquired microcephaly. Choreoathethosis and ataxia are described in infantile-onset cases [52].

*GABRB3* (gamma-aminobutyric acid receptor, Beta-3): Among 22 cases of epilepsy/EE (with mean onset at 8.7 months), one patient experienced neonatal-onset EE. His seizure types are polymorphous (focal, tonic-clonic, spasms). He has severe DD and experienced stagnation aged 3 months [53].

*GABRG2* (gamma-aminobutyric acid receptor, gamma-2): In a cohort of 5 patients harbouring a recurrent c.316G>A; p.A106T pathogenic variant, two had intractable, neonatal-onset epilepsy (not further characterized: one; ictal apnea: one), followed by focal versive, myoclonic and tonic-clonic seizures associated with hypotonia, severe DD, cortical visual impairment, and stereotypies in one [60].

*GRIA2* (glutamate receptor, ionotropic AMPA 2): 2/24 patients (8%) with neurodevelopmental disorder and EE had neonatal-onset seizures and MD. Seizures at onset were tonic hypomotor, with subtle phenomena and myoclonic jerks or eyelid myoclonia. Subsequent seizure types included focal and ES. Neonatal EEG showed a normal background with multifocal discharges, later evolving into high-voltage-slow. Neurological examination revealed hypotonia, DD and decelerating head growth, or acquired microcephaly. The associated neonatal MD is characterized by non-epileptic myoclonus and exaggerated startle, while, at 3 years of age, one patient is described as hypokinetic with oculogyric crises [54].

*GRIN2B* (glutamate receptor, ionotropic, N-Methyl-D Aspartate, subunit 2B): The only patient with neonatal-onset epilepsy had subtle seizures, followed by spasms. He has severe DD and a dyskinetic MD. Brain MRI is normal. No detailed information is available on neonatal EEG or subsequent epilepsy course. The mean age at epilepsy onset in the cohort is 6.5 years, and the core phenotype is represented by a neurodevelopmental disorder featuring hypotonia, MD (choreo-athethosis, dystonia), cortical visual impairment and cerebral volume loss [58].

#### 3.4.9. G-PROTEIN Transduction

*GNAO1* (guanine nucleotide-binding protein, alpha-activating activity polypeptide O): *GNAO1* encephalopathy clinically encompasses a complex neurologic phenotype associating severe and disabling hyperkinetic MD with/without EE. Neonatal-onset EE occurred in 7/26 of reviewed cases, typically with tonic, tonic and clonic seizures, or spasms, although focal seizures with apnea (later evolving into focal hypomotor with dysautonomic features) were also reported. EEG is severely abnormal with multifocal discharges or burst-suppression. Six patients developed an MD (dystonic in three, dyskinetic or choreic in two each, akathisia in one) and, during the follow-up, severe-to-profound DD, swallowing difficulties, and sometimes microcephaly [15,16,17,18,19].

#### 3.4.10. Neuronal Connectivity/Signal Transduction

*PCDH12* (protocadherin 12): 3/4 in a consanguineous family had neonatal-onset focal, tonic seizures or spasms. EEG showed variable findings (focal, multifocal or hypsarrhythmia). They had visual impairment, hypotonia and microcephaly, and profound DD. Congenital dystonia was reported in 3/4 cases [45].

#### 3.4.11. Ubiquitination

*RHOBTB2 *(rho-related BTB domain-containing protein 2): infantile-onset DE/EE and MD (chronic and/or paroxysmal) in the context of severe DD are the main clinical features. One out of ten patients has been reported with “generalized” neonatal-onset epilepsy. MD is complex, incorporating chorea and dystonia, together with paroxysmal dyskinetic attacks. Epilepsy outcome is usually good, as it was in this neonatal-onset case, while MD is ongoing [57].

#### 3.4.12. Transporters

*SLC13A5* (solute carrier, family 13, member 5): Patients present a consistent phenotype: neonatal-onset EOEE with clonic and/or subtle seizures and a high rate of status epilepticus. Ictal EEG is characterized by multifocal discharges with predominant temporal or temporo-occipital foci. A high proportion of cases (5/7 in [28]) have complex MD, with ataxia, choreo-athethosis, dystonia, and/or dyskinesia. Age of onset is not reported. Variable DD is always present. Microcephaly can be a feature. The most consistent additional report is hypodontia or teeth hypoplasia. White-matter changes can be reminiscent of periventricular leukomalacia [30].

## 4. Discussion

Although more than 100 genes have been associated with complex monogenic neurologic disorders featuring pediatric-onset DE/EE and MD [61,62,63], this association has been less investigated in newborns. As a result, the number of reviewed studies is low and, for 70% of genes, only single papers were available.

This likely reflects our search strategy’s exclusion of neurometabolic disorders. In fact, the association between epilepsy/EE and MD in newborns should raise suspicion of neurometabolic disorders, especially mitochondrial [7] or neurotransmitters disorders (although highly heterogeneous) [64,65]. While biomarkers can help diagnostic work-up in these conditions [66,67], this is not the case with genetic diagnoses, where accurate semiological, EEG and neuroimaging data evaluations become imperative.

This review highlights that, although singularly rare, the number of disorders with possible neonatal onset is high, with 27 different genes involved, all playing key roles in cellular functions critical for brain development from its earliest stages [68]. The most conspicuous groups are neurotransmitters receptors, ion channels and synaptic formation and function. From a clinical standpoint, this translates into disrupting crucial neurodevelopmental trajectories, resulting in complex neurodevelopmental disorders featuring epilepsy/EE-DE, MD, but also DD or ID developing in all reviewed conditions. Furthermore, a high rate of microcephaly and/or decelerating head growth is present, with frequent co-occurrence of autistic features, adding evidence that neurodevelopmental disorders have a strong genetic component and comprise a range of co-existing morbidities [68,69]. Furthermore, as can be usefully exemplified by *CDKL5*, the complex interplay with other gene products, i.e., *MECP2 (*methyl CpG binding protein 2), which works as a transcriptional repressor/activator, results in partially overlapping, complex neurodevelopmental phenotypes falling within the so-called Rett syndrome spectrum [70]. None of the patients with monogenic disorders presented a fully “benign” phenotype; even though, in some, a partial or transient seizure control was achieved.

Although we searched for both “epilepsies” and “DE-EE”, this population is enriched with EE and mainly falls into an EOEE diagnosis. Therefore, the prevalent seizure types in the neonatal period are tonic or myoclonic. With recent advances in genetic testing, there has been renewed interest in the relationship between seizure semiology and etiology in newborns, documenting higher occurrence of myoclonic and “sequential” seizures with burst-suppression in genetic aetiologies [71]. An important exception is represented by *SCN2A* cases, in which BFIS are accompanied by no or mild DD, seizure freedom since infancy, and are followed by the development of childhood-onset episodic ataxia.

As far as MD is involved, the majority of patients develop chronic hyperkinetic disorders. Age of onset is often lacking, but it falls within the neonatal period only in a minority of cases, with myoclonus or myoclonus-like dyskinesia, dystonia (including opistotonus), athethosis, tremors, or startle. Only in patients with *WWOX* gene variants there is an associated early onset hypokinetic MD with little or no psychomotor development from birth [46]. In the remaining cases, hypokinetic MD develops later in the disease course: in early childhood in *GRIA2*-related disorder, during teenage years in *STXBP1*, with parkinsonism [33,34]. However, as hyperkinetic MD might be easier to recognize, a potential bias cannot be completely excluded.

Neuroimaging is often unremarkable during the neonatal period, but, afterwards, progressive cerebellar or cerebral degenerative changes can be detected [6,15,16,17,18,19,43,51,52,53,54,55].

As in the majority of cases, MD onset occurs during infancy or childhood, and neonatal-onset MD can be useful to prioritize differential diagnosis: neonatal-onset hypokinetic MD appears exceptional and highly suggestive for *WWOX*-related disorders. Neurometabolic conditions being ruled out, hyperkinetic neonatal-onset MD should first raise suspicion of an *SCN8A* or *STXBP1*-related disorder, as these are associated with myoclonus/myoclonus-like dyskinesia (*STXBP1*, but also reported in *KCNQ2*), dystonia and/or opistotonus (*SCN8A*, although also described in *PCDH12*), tremors (both conditions), and startles (both, plus *GRIA2*). Complex paroxysmal episodes of dystonic-tonic posturing with eye movement abnormalities should raise suspicion of an *ATP1A3*-related disorder, while paroxysmal tonic episodes alternating with hypotonia have been documented in *SCN8A*. Outside these conditions, data to set up the diagnostic process since the first days of life mainly pertain to the epilepsy phenotype, in some cases assisted by neuroimaging data. However, as already discussed, the majority of patients will present with EOEE, often characterized by burst-suppression or a multifocal pattern and tonic/myoclonic seizures with limited specificity, as only *KCNQ2* encephalopathy has been robustly associated with a typical seizure type. In clinical practice, this lack of specificity results in a high priority of NGS panels or whole exome sequencing in the diagnostic work-up.

We documented a high rate of missense variants [72,73], reflecting the notion that these can be frequently deleterious in humans and contribute to complex disorders [74]. For AD conditions, it is clinically relevant to highlight that, despite the vast majority of variants causing severe EE/DE phenotypes occurring de novo, parental mosaicism should always be considered and risk assessment with high depth coverage offered to parents.

Detailed information about neonatal ictal semiology and EEG findings is missing more often than in older age groups, highlighting the need for better characterization of ictal semiology and neonatal ictal, as well as interictal EEG characteristics in this age group.

Although precision medicine is a clinical practice in only a few conditions (*KCNQ2*, *SCN2A*, *SCN8A*), it is mandatory to improve our knowledge of early presenting features. To this end, lines of future research should also focus on defining age ranges of onset, clinical features, evolution, and therapeutic strategies for the associated MD, as these are often less discussed compared to epileptic phenotypes.

## Figures and Tables

**Table 1:** Function of genes involved in neonatal-onset epilepsies/EE and MD.

Functional Role	Gene Name
Channelopathies	*CACNA1E*, *KCNQ2*, *SCN2A*, *SCN8A*
ATPase	*ATP1A3*
Synaptopathies	*AP3B2*, *DNM1*, *STXBP1*, *VAMP2*, *SYNJ1*
G protein transduction	*GNAO1*
Transcription factors	*FOXG1*
Transcription regulators	*CDKL5*, *SMC1A*, *PURA*
Neuronal connectivity/signal transduction	*PCDH12*
Ubiquitination	*RHOBTB2*
Receptors	*GABRA1*, *GABRB2*, *GABRB3*, *GABRG2*, *GRIA2*, *GRIN2B*
Enzymes	*WWOX*
Growth factors	*FHF1*
Transporters	*SLC13A5*

**Table 2:** Electroclinical syndromes according to genetic etiology.

Electroclinical Phenotype	Gene Name
EOEE (22/27; 81.5%)	*AP3B2*, *ATP1A3*, *CACNA1E*, *CDKL5*, *DNM1*, *FOXG1*, *FXF1*, *GABRA1*, *GABRB2*, *GABRB3*, *GABRG2*, *GNAO1*, *KCNQ2*, *PCDH12*, *RHOBTB2*, *SCN2A*, *SCN8A*, *SLC13A5*, *SMC1A*, *STXBP1*, *SYNJ1*,*WWOX*
BFIS (1/27; 3.7%)	*SCN2A*
KCNQ2-RELATED ENCEPHALOPATHY (1/27; 3.7%)	*KCNQ2*
NOT CATHEGORIZED AS EE/DE (4/27; 14.8%)	*GRIA2*, *GRIN2B*, *KCNMA1*, *PURA*, *VAMP2*

**Table 3:** Movement disorder semiology according to genetic etiology.

Type of MD	Gene Name
HYPERKINETIC MD	
Ataxia	*FHF1 KCNQ2 SCN8A STXBP1 SLC13A5*
Dystonia	*AP3B2 CACNA1E DNM1 GABRB2 GNAO1 KCNQ2 PCDH12 RHOBTB2 SCN2A SCN8A STXBP1 WWOX SLC13A5 SYNJ1*
Status dystonicus	*GNAO1*
Stereotypies	*AP3B2 CDKL5 FOXG1 KCNQ2 GABRG2 SCN2A SMC1A STXBP1*
Tremor	*KCNQ2 SCN8A STXBP1*
Chorea	*CACNA1E GNAO1 KCNQ2 (with fever) RHOBTB2 SCN2A STXBP1 VAMP2 SLC13A5*
Choreo-athethosis	*GABRA1 STXBP1 SLC13A5*
Athethosis	*ATP1A3 PCDH12*
Dyskinesia	*AP3B2 CACNA1E FOXG1 GABRB3 GNAO1 SCN8A STXBP1 SLC13A5 KCNQ2 (myoclonus-like)*
Akathisia	*GNAO1*
Myoclonus	*CACNA1E GABRA1 GRIA2 KCNQ2 SCN8A STXBP1 WWOX*
Oculogyric crises	*GRIA2 SCN2A*
Paroxysmal dyskinesia	*KCNMA1 RHOBTB2*
Episodic ataxia	*SCN2A*
Paroxysmal non-epileptic polymorphous events	*ATP1A3 SCN8A*
Paroxysmal involuntary movements	*WWOX*
Startle/hyperekplexia	*GNAO1 SCN8A STXBP1 WWOX*
HYPOKINETIC MD	
Bradykinesia	
Hypokinesia	*GRIA2 WWOX*
Hypokinetic-rigid syndrome	*STXBP1*
UNSPECIFIED	
PURA	

**Table 4:** Brain MRI findings according to genetic etiology.

Brain MRI Findings	Genes
NORMAL	*CACNA1E*, *CDKL5*, *DNM1*, *FXF1*, *GABRA1*, *GABRG2*, *GNAO1*, *GRIN2B*, *KCNMA1*, *KCNQ2*, *PURA*, *SCN8A*, *SLC13A5*, *STXBP1*, *VAMP2*, *WWOX*
Cerebral atrophy	*ATP1A3*, *CACNA1E*, *FOXG*, *GABRA1*, *GNAO1*, *KCNQ2*, *SCN8A*, *STXBP1 SYNJ1*
White matter abnormalities (including hypomyelination)	*CACNA1E*, *GABRB2*, *GABRB3*, *GRIA2*, *KCNQ2*, *SCN2A*, *SLC13A5 SYNJ1 (*periventricular *WM* gliosis*)*
Cerebellar atrophy	*FHF1*, *GABRA1*, *GRIA2*
Corpus callosum hypoplasia	*FOXG1*,*GABRA1*, *WWOX*
Thin CC	*AP3B2*, *CACNA1E*, *GNAO1*, *KCNQ2*, *SMC1A*, *SYNJ1*, *WWOX*
Myelination delay	*ATP1A3*, *CACN1E*, *FOXG1*, *GNAO1*, *RHOBTB2*, *SCN8A*
Basal Ganglia hyperintensities	*ATP1A3* (GP*)*, *CACNA1E*, *GNAO1* (GP), *KCNQ2 (BG and THALAMI*, neonatal age), *SCN2A*
Cortical malformations	*FOXG1*, *KCNQ2* (simplified gyral pattern)
Cerebellar hypoplasia	*GRIA2* (vermis)
Enlarged extra-axial space	*AP3B2*, *WWOX*
Brainstem hyperintensities	*SCN2A*
Midbrain, hypothalamus and optic trait dysplasia	*PCDH12*
Small frontal lobes	*SMC1A*
Small thalami	*KCNQ2*

## Supplementary Materials

The following are available online at https://www.mdpi.com/article/10.3390/ijms22084202/s1. Table S1: Data collection sheets with detailed information from the 49 included articles.
