# Supplementary material for: Genetic Neonatal-Onset Epilepsies and Developmental/Epileptic Encephalopathies with Movement Disorders: A Systematic Review
Source: Int J Mol Sci. 2021 Apr 18;22(8):4202. doi: 10.3390/ijms22084202 (PMC8072943; doi:10.3390/ijms22084202)
Supplement: Supplementary file 1 [file ijms-22-04202-s001.zip › Table S1.pdf]

Gene	Disease name, #OMIM	design	Oxford Centre for EBM level of evidence	n (gender)	Mode of Inheritance	Gene Variant(s)	Impact on transcript	Functional studies	ACMG criteria	Segregation analysis	Method of ascertainment	E onset (age)	seizure semiology	EE/DE	E offset (age)	Neonatal EEG	Follow-up EEG	Additional findings	DD	Behavioural/ psychiatric features	stagnation/ regression/ progressive symptoms	MD semiology	MD onset (age)	MD offset (age)	Brain MRI	Last F/U (age)	Ref
AP3B2	EIEE48, OMIM #617276	OA	4	12<br>2/12	AR	homozygous: c.2522_2525delTCAC<br>c.1110 p.1G>C	frameshift<br>skipping of exon 9	Performed (only in families 1 and 2)	LP	heterozygous carrier parents	WES	Birth-1 mo	Subtle NS: 1<br>T: 1<br>SE (1mo): 1	EOEE	Resolved: 1/2 (age: NA)	NA	NA	Hypotonia: 2/2<br>Reduced/absent DTRs: 2/2<br>Microcephaly: 2/2<br>Optic nerve pallor: 1/2	Severe/profound DD:2/2<br>Absent speech: 2/2	-	(Regression, infantile -onset)	Stereotypies: 2/2<br>Dyskinesia: 2/2<br>(Choreo-athethosis, infantile onset)	NA	NA	N: 1<br>thin CC, enlarged extra-axial space: 1	32mo; 24mo	38
ATP1A3	alternating hemiplegia of childhood, OMIM#614820	CS	4	2 (1F)	AD	c.1073G>T, p.Gly358Val	In-frame deletion	performed	LP	De novo	NGS	4h	Onset: NA<br>Follow-up: Todd paralysis	EOEE	-	NA	Multifocal seizures	postnatal microcephaly	DD	-	-	episodes of nystagmus, disconjugate gaze, mouth and tongue movements, and reduced responsiveness<br>continuous athetotic movements	2mo<br>11mo	.	progressive<br>atrophy	16mo (died)	13
ATP1A3	alternating hemiplegia of childhood, OMIM#614820	CR	4	1 M	AD	c.2736_2738CTTdel, p.Phe913del	?	Not performed	?	De novo	WES	D2	NA	«Catastrophic early-life epilepsy»	-	Focal (multifocal ictal events, non-migrating)	NA	Extreme hypotonia<br>microcephaly<br>feeding difficulties<br>respiratory and cardiac failure	Profound DD	-	-	Non-epileptic tonic and dystonic events, episodic oculomotor abnormalities with tachycardia	D2	-	High GP intensity bilaterally (D6)<br>delayed myelination (10mo)<br>progressive atrophy (2y and 4y)	4y	14
CACNA1E	EIEE69, OMIM #618285	R OA	4	33 (19F), unrelated	AD	c.683T>C c.1042G>C c.1054G>A in 9 c.1807A>C c.2069G>A c.2093T>C c.2098G>A c.2101A>G in 3 c.2104G>A in 6 c.2104G>C c.4264A>T c.4274C>A in 2 c.4288G>A c.5159C>G	Missense n= 30<br>premature termination codons: 3	performed	P	De novo except for 2 cases:<br>n=1 parents unavailable<br>n= 1 mother tested negative, father unavailable	Trio WES + Sanger validation (duo WES performed in 1) OR<br>Research-based trio WGS<br>OR NGS targeted panel analysis	Mean 8 mo (range : D1-3y)	Whole cohort :<br>Multiple: 21/30 ES: 16/30 (53.3%) FM: 10/30 (33.3%) T: 9/30 (30%) Myo: 8/30 (26.7%) GTCS: 6/30 (20%) fias: 6/30 (20%) SE : 2/30 (6.7%) gel: 1/30 (3.3%) At : 1/30 (3.3%)<br>Neonatal: ES 2<br>Myo : 2	26/30	-	MFD: 15/30 (50%)<br>Hyps: 8/30 (26.7%)<br>CSWS, FD, Slow SW, B-S: 1/30 (3.3%) each<br>N.A.: 3/30 (10%)		Prominent axial hypotonia: 100% spastic quadriplegia: 16/30 (53%)<br>Congenital joint contractures: 13/30 (43%)<br>Macrocephaly: 12/28 (43%)<br>Early death: 7 (23%) median age 2.7 y (range 11mo-25y)	DD: profound 26/30 (86.6%) Severe 3/30 (10%) unspecified: 1/30 (3.3%)<br>Of the probands > 2 y: 21/24 (88%) non-verbal & non-ambulatory	-	Developmental regression : 9/30 (30%)	Dystonia : 12/30 (40%)<br>Chorea: 3/30 (10%)<br>Dyskinesias: 2/30 (6.7%)<br>Myoclonus: 1/30 (3.3%)<br>Unknown: 1/30 (3.3%)<br>“Hyperkinetic”: 1/30 (3.3%)<br>Multiple: 4/30 (13.3%)	Early-onset (unspecified)	-	N: 19/30 (28.9%), WM loss: 2/30 (6.7%), Cortical atrophy: 4/30 (13.3%), WM hyperintensity, BG hyperintensity, Delayed myelination, Thin CC, NA: 1/30 (3.3%) each	Mean: 6.3 y (range: 10 m-25y)	35

CDKL5	EIEE2,<br>OMIM<br>#300672	P OA	4	92 Rett/Rett-like<br>17 Angelman-like<br>NE + MD: 2/7	XLD	c.903_904dupGA, p.Leu302Aspfx49X<br>c.2376+5G>A	Frameshift<br>Abolishing splice site	Not performed	P<br>LP	De novo: 2/2	Sequencing + MLPA	2w; 3w	Onset:<br>Myo: 1<br>F: 1<br>Evolution:<br>F with impaired awareness: 1 F myo: 1	EOEE: 2/2	Ongoing: 1<br>Seizure-free (6mo): 1	NA	Slow: 1<br>MFD: 1<br>Unspecified ED: 1	Hypotonia: 2/2<br>Head deceleration: 2/2<br>Scoliosis: 1/2<br>Autonomic features: 1/2	Severe ID: 1/2<br>Mild: ½<br>Ambulant: 1/6<br>Absent speech: 1/2	Autistic features: 1/2<br>Eye contact absent/inconstant: 2/2<br>p.Leu302Aspfs X49 → Angelman phenotype<br>c.2376+5G>A → Atypical Rett	n= 1, age NA	Hand stereotypies: 2/2	NA	-	N: 1<br>NA: 1	7mo; 6y	41
DNM1	EIEE31, OMIM #616346	R OA	4	21 (7 pu)<br>NE + MD: 1/21	AD	c.127G.A, p.Gly43Ser	missense	Not performed (in silico prediction models used)	P	De novo	NGS panel/WES	3w	Myo<br>Ab	DEE	Seizure-free (6y, LEV + LTG)	nonspecific background slowing		Microcephaly<br>spasticity<br>hypotonia	profound DD<br>(non-verbal<br>non-ambulant)			Dystonia	NA	NA	N	8y	43
FOXG1	Rett syndrome, congenital variant, OMIM #613435	OA	4	45 (23F)<br>MD: 45/45 (100%)<br>E + MD: 35<br>NE + MD: 1	AD	del14q12 (18,798,641–19,484,013)	CNV	Not performed	P	De novo	array CGH	D2	F	-	< 1 FS/month	NA	NA	Limited hand movement	DD	-	-	generalized hyperkinetic<br>dyskinetic<br>orolingual<br>hand stereotypies	-	-	Mild SIMP gyral pattern moderate cortical atrophy, myelination delay, hypoplastic CC	8y	44
FGF12 (FHF1)	EIEE47, OMIM #617166	OA	4	2 related (1M)	AD	c.155G.A, p.R52H	missense	performed	P	De novo (suspected germline mosaicism)	WES	2w; 4w	T: 2<br>SE	EOEE	-	severe background slowing<br>MFD<br>Ictal: low voltage fast activity, followed by long suppression	Hyps (without IS): 1 (5mo)	Hypotonia: 2<br>Microcephaly: 2<br>Feeding difficulties: 2<br>pale optic discs	Profound GDD (non-ambulant, non-verbal)	-	Regression following sz onset (N prior to sz onset)<br>Degeneration (decrease in alertness, general condition, and skills)	Limbs Ataxia: 1<br>Ataxia: 1	NA	-	N (2mo; 5mo)<br>Cerebellar atrophy (3y; 6y)	Died: 7y (SE); 3.5y	37
GABRA1	EIEE19, OMIM #615744	OA	4	6<br>NE + MD: 2/6	AD	c.788T>C (p.M263T)<br>c.859G>T (p.V287L)	missense	Not performed (in silico prediction models used)	P : 2/2	De novo	WES (546)<br>GABRA1 targeted resequencing (145)	Mean: 2.6mo (range : Birth-10mo)	Onset<br>Myo: 1<br>T: 1<br>Evolution<br>WS: 1<br>EOEE: 1	EOEE	Refractory: 1<br>GBP: 1 (Age NA)	Trace alternant with MFD: 1<br>Diffusely slow with MFD: 1	Hyps with MFD: 1<br>B-S: 1	Acquired microcephaly: ½<br>NA: ½<br>Hypertonia: 1/2	Profound ID	-	-	Myoclonus: 1<br>Choreathethosis: 1	NA<br>NA	-<br>2y	N (birth) - Cerebral atrophy (3mo): 1<br>Sovra+Sub-tentorial atrophy, thin CC (4 y)	10y: 1<br>died at 1y: 1	45
GABRB2	EIEE2, OMIM #617829	P OA	4	6<br>NE + MD: 1/6	AD	c.908A>G (p.Lys303Arg)	missense	Not performed (in silico prediction models used)	P	De novo	Targeted NGS panel	D1	T, F, MF	EOEE	Ongoing (improved on TPM)	MFD<br>Slow background	NA	Microcephaly<br>Hypotonia<br>feeding difficulties	GDD<br>Severe ID	-	-	dystonia	NA	NA	WM T2 hyperintensity	7 y	46
GABRB3	EIEE43, OMIM #617113	R OA	4	E + MD: 7/22<br>NE + MD: 1/22	AD	c.767T.A, p.L256Q	missense	Performed	P	De novo	Massive parallel sequencing	D1	F GTC IS	EOEE	pharmacoresistant	Hyps<br>GD		Hypotonia	Severe DD		Stagnation: 3mo	Dyskinesia: 1	NA	NA	Hypomyelination	NA	47
GABRG2	EIEE74, OMIM #618396	R CS	4	5, unrelated (3F)<br>NE + MD: 1/5	AD	p.A106T (c0.316G>A)	missense	Not performed (in silico prediction models used)	P	De novo	NGS panel	D1	NS (not defined)<br>Myo<br>F versive with impaired awarenes	EOEE	Response to LEV + OXC (age NA)	unremarkable	Excess Beta activity<br>Frontal sharp	CVI<br>Hypotonia<br>Dysmorphic features	Severe (non-ambulatory, verbal: NA)	Lack of social smile, poor visual interaction	-	Continuous stereotypies	NA	-	N	13y	55

													s														
GRIA2 (GluA2)	OMIM#138247	OA	4	28 pts<br>E + MD: 7/28 (4F)<br>NE + MD: 2/24	AD	c.2363G>T<br>p.Trp788Leu<br>c.1915G>T p.Ala639Ser	missense<br>missense	Performed	LP: 2/2	De novo: 2/2	WES	Birth; D7	Onset<br>T hypomotor<br>Myo eye blinking and mastication, +/- hypertonia and bradycardia<br>Follow-up<br>ES<br>F	DEE	-	N background: 2<br>MFD: 1	MFD<br>High voltage slow background	Deceleration of head growth:1<br>Microcehaly:1<br>Axial hypotonia: 2	yes	-	-	myoclonus in the face and limbs Exaggerated startle response: 1<br>Hypokinetic: 1 Oculogyric crises: 1	Neonatal<br>3y	-	Cerebellar atrophy: 1<br>Cerebellar vermis hypoplasia: 1<br>hypo-myelinating leukodystrophy: 1	3.5y<br>3mo (died of SUDEP; p.Ala639Ser)	51
GNAO1	EIEE17, OMIM #615473	OA	4	14<br>NE + MD: 3/14	AD	c.119G>A (p.G40E)<br>c.818A>T (D273V)<br>c.836T>A (p.I279N)	Missense: 3/3	Not performed (in silico prediction models used)	P: 3/3	De novo: c.836T>A; c.818A>T<br>Suspected parental germline mosaicism (2%, below reporting threshold) for c.119G>A	Targeted NGS panel<br>WES	Neonatal: 3 (1h, 2h, D2)	Onset<br>"T-C": 2<br>ES: 1<br>Evolution:<br>IS: 1<br>F: 1<br>NA: 1	DEE: 3/3	-	MFD: 2<br>high amplitude bursts: 1<br>Abn: 1<br>IED: 1	Mod Hyps (8mo): 1<br>Sev abn: 1<br>FD: 1	Hypotonia: 3/3<br>Scoliosis: 1<br>Swallowing dysfunction: 1	ID: 3/3<br>Non-ambulant, non-verbal: 2/3	-	Yes (stagnation)	Focal dystonia: 1<br>Dyskinesia: 1<br>Chorea: 1<br>Akathisia: 1<br>(multiple: 2)	12 mo: 1<br>NA: 4/5	NA	N: 1<br>Increased GP signal: 1<br>Delayed myelination: 1<br>Atrophy: 1	2; 6; 15y	15
GNAO1	EIEE17, OMIM #615473	CR	4	1F	AD	c.596T>C p.Leu199Pro	missense	Not performed (in silico prediction models used)	P	De novo	Trio-WES	neonatal	T/subtle	EOEE	Seizure-free on KD (age NA)	Sev abn background<br>MFD	Sev abn background with poor differentiation and no sleep elements + bilateral IED	Severe axial hypotonia<br>Microcephaly<br>Poor eye fixation	Severe GDD	-	-	Dyskinetic bucco-lingual movements<br>Reduced spontaneous movements<br>startle	21mo<br>3mo<br>3mo	-	Mild cerebral atrophy, thin CC, delayed myelination	21mo	19
GNAO1	EIEE17, OMIM #615473	OA	4	4 pts<br>NE + MD: 1/4	AD	c.607G4A p.(Gly203Arg)	missense	Not performed (previously described)	P	De novo	WES + Sanger sequencing	D7	Onset<br>"T-C"<br>Evolution<br>F with impaired awareness	EOEE	refractory	Slow-wave bursts,<br>Migrating FD	MFS<br>Asymmetry	-	Profound DD	-	--	Severe chorea	NA	-	N (D20)<br>Progressive cerebral atrophy + delayed myelination (14 mo)	14mo	16
GNAO1	EIEE17, OMIM #615473	R OA	4	4 F<br>NE + MD: 1/4	AD	c.572_592 del p.Thr191_Phe197 del	frameshift	Performed	P	De novo	WES + mutation screening	2w	T	EOEE<br>(OS)	refractory	B-S	Hyps (4mo)	-	Profound DD		-	Dystonia	NA	-	N (3mo)	11mo (died)	17
GNAO1	EIEE17, OMIM #615473	OA	4	4 pts (3F)<br>NE + MD: 1/4	AD	c.607G > A p.Gly203Arg	missense	Not performed ((in silico prediction models, additional descriptions)	P	De novo	WES	D9	Onset<br>F with apnoea<br>Evolution<br>T, T-C, hypomotor autonomic	EOEE	Effective LTG+ZNS (age NA)	abn	Ictal T discharges	Hypotonia<br>Dysphagia (PEG-fed)	DD	-	-	Dystonic movements	NA	Gabapentin, trihexyphenidyl: no effect; BDZ: reduced paroxysmal dystonia (age NA)	N	4y	18
GRIN2B	EIEE27, OMIM # 616139	R	4	39 N<br>E + MD: 6	AD	c.2065G>A p.(Gly689Ser)	missense	Performed	P	De novo	NGS panel	D1	Onset<br>Subtle sz<br>Evolution:	WS	-	NA	FD	Hypertonus	Severe ID	ASD: 2	4/58<br>(MD:	Dyskinetic	NA	NA	N	1y	53

				NE + MD: 1/6									ES								1/6)						
KCNMA 1	cerebellar atrophy, developmental delay, and seizures, OMIM #617643		4	2<br>NE + MD: 1/2	AD	c.2650G>A p.Glu884Lys<br>c.3158A>G p.Asn1053Ser	missense	Not performed (in silico prediction models used)	LP c.2650G>A<br>P: c.3158A>G	De novo	Targeted NGS panel	D20	NA	NA	-	N		????	DD<br>Severe	NA	NA	PNKD	NA	NA	N		54
KCNQ2	EIEE7, OMIM # 613720	R Case series	4	NE + MD: 2/2<br>(1F)	AD	c.619C>T p.Arg207Trp	missense	Not performed	P	Maternally inherited	Direct sequencing	D2	Clonic + desaturation: 1<br>Focal tonic: 1	Familial neonatal-onset epilepsy	Neonatal period offset: 1<br>12y: 1 (on LTG)	Bilat background slowing (adult): 1 Rolandic discharges (newborn): 1		Myokymia of the abdominal wall muscles	GDD (language most affected): 1<br>ID: 1	Reduced attention span	-	Paroxysmal "myoclonus-like" dyskinesia (limbs, eye, diaphragm, limbs, vocal cord)<br>ataxic gait, hyperkinetic dyskinesia, mild distal tremor<br>"myoclonus-like" dyskinesia in her limbs: 1	3w<br>2y<br>NA	-	N MRI: 1<br>Bilaterally open operculum, irregular gyral pattern, small F lobes, abnormal extra-axial fluid quantity: 1	28 mo: 1<br>Adulthood, unspecified: 1	22
KCNQ2	EIEE7, OMIM # 613720	CR	4	1M	AD	c.619C>T (p.R207 W).	missense	Not performed	P	De novo	Trio WES	D7	F	KCNQ2-DEE	Neonatal period offset (OXC)	NA	N	hypotonia	mild	NA	-	Chorea and myoclonus during febrile illnesses	NA	Cessation with fever resolution	N	8y	24
KCNQ2	EIEE7, OMIM # 613720	OA	4	NE + MD: 1/8 (M)	AD	c.1636A>G p.Met546Val	missense	Not performed	P	De novo	Direct sequencing	D3	T + hemiclonic + eyelid myoclonia	KCNQ2-DEE	3y	B-S	MFD (3w)<br>N (8y)	Mild spasticity<br>dismorphisms<br>hypospadia	DD, non-verbal	autism	-	Hand stereotypies: 1<br>broad-based gait	NA	-	Whole cohort: T1 and T2 BG and thalami hyperintensities (less obvious with decreasing age)	9y	20
KCNQ2	EIEE7, OMIM # 613720	OA	4	NE + MD:4/11 (3F)	AD	c.943G>C, p.Gly315Arg<br>c.973A>G, p. Arg325Gly<br>c.1655A>C, p.Lys552Thr<br>c.1687G>A, p.Asp563Asn	missense	Not performed (in silico prediction models used)	P: 4/4	De novo	Sanger direct sequencing<br>NGS panel	D1: 3<br>D3: 1	T, apnoea, desaturation, bradychardia, desaturation, chewing	KCNQ2-DEE	1 sz-free (10 mo)	B-S: 3<br>FD: 1	Slow background: 3<br>N: 1<br>MFD: 4	Axial hypotonia	Profound DD: 2<br>Mild/Mod: 1<br>Severe ID: 1	Autistic features: 1	-	Opistotonus: 1<br>Dystonic posturing: 3<br>Hand stereotypies: 2	NA	-	F lobes atrophy: 2<br>reduced WM: 2<br>thin CC: 2<br>small thalami: 2 (11mo; N at 4mo; 3w)<br>dilated ventricles: 2	NA	21
KCNQ2	EIEE7, OMIM # 613720	CR	4	1M	AD	c.913_915del [p.Phe305del)]	Intragenic deletion	Not performed (additional descriptions)	P	De novo	NGS panel	10h	F T + Tonic spasms	KCNQ2-DEE	2mo (CBZ)	B-S	Abnormal<br>MFD	Pyramidal syndrome	Profound DD	-	-	dystonia	9mo	.	Thin CC	9mo	23
PCDH12	diencephalic-mesencephalic junction dysplasia syndrome 1<br>OMIM<br>#251280	Case series	4	n= 4<br>3 (2F) from 3 consanguineous families<br>E + MD: ¾<br>NA: 1/4	AR	Biallelic c.2515C>T p.Arg839 (4)	nonsense	Performed	P	Parents heterozygous carriers	WES + homozygosity mapping	Mean: D10.6 (range: D4-D21)	F: 2<br>T:1<br>IS: 1<br>Multiple types:	1/3 (IS)	-	Hyps: 1/3 MFD: 1/3 FD: 1/3		Axial hypotonia: 3/3 VI:3/3<br>Progressive microcephaly: ¾ (NA: ¼)	profound	No acquisition	-	Dystonia: 3/3	Congenital	-	Midbrain hypothalamus- optic tract dysplasia: 3/3	16.6 y (range: 3-26)	39

PURA	mental retardation, autosomal dominant 31, OMIM #616158	OA + lit R/w	4	30<br>NE + MD: 1/30	AD	c.812_814del p.(Phe271del)	Intragenic deletion	Performed	P	De novo	WES	neonatal	myoclonic	NA	NA	Abnormal (not described)	NA	Hypotonia	Mod ID (non-ambulant, non-verbal)	-	-	NA	NA	NA	N	23 mo(last examined at 11mo)	49
RHOBTB2	EIEE64, OMIM #618004	R	4	10 (6F)<br>E + MD: 9/10 (90%)<br>NE + MD: 1/9	AD	c.1448G>A	missense	Performed	P/LP	De novo	Trio WES	Range: D4-3y (mean: 5m)	"Generalized"	yes	Yes age NA	NA		Hypotonia<br>LL's hypertonia<br>dysmorphisms	Severe			Chorea Dystonia<br>Paroxysmal dyskinetic attacks<br>Multiple types: 1	NA	NA	Delayed myelination	3.5y	52
SCN2A	DEE11, OMIM #613721<br>BFIS3, #607745<br>EA9, #618924	R OA	4	12<br>EE + MD: 10 (5F)<br>NE + MD: 7	AD	c.4777G>A c.2715G>C c.5645G>A c.4901G>T c.653C>A c.718G>T<br>c.2567G>T (mosaic, 34% mutant load in lymphocytes)	missense	Not performed	P: c.4777G>A c.2715G>C c.2567G>T<br>LP: c.5645G>A c.4901G>T c.718G>T c.653C>A c.2567G>T	De novo: 6/7<br>not maternal, paternal DNA NA (c.653C.A)	Targeted sequencing with molecular inversion probes<br>WES	21.95 mo (0-17y)	F (multiple types): 7<br>T: 1<br>ES: 2	EE: 7/7<br>EIMFS: 5 OS:2	2/7<br>(3mo, 3.5mo)	MFD: 7/7<br>BS: 2/7<br>N/slow: 5<br>N: 1		Axial hypotonia: 6<br>Appendicular hypertonia: 4	Mild/mod DD: 1<br>Sev/Prof: 5<br>N: 1<br>Mild LD: 1	Autistic features: 1	none	Stereotypies: 1 Oculogyric crises: 2 Opisthotonus: 1 Dystonia: 4 Chorea: 1 Multiple: 2	-	-	WM involvement: 7<br>BG: 6<br>Brainstem: 2<br>NA: 1		36
SCN2A	DEE11, OMIM #613721<br>BFIS3, #607745<br>EA9, #618924	R	4	4<br>NE + MD: (3M)	AD	c.5644C>G<br>c.5644C>G c.4565G>C (in cys)<br>c.788C>T in 2	Missense	Yes (whole-cell patch-clamping in mammalian cells)	P	De novo, except c.4565G>C (paternal origin)	Direct sequencing (1 pt), NGS epilepsy panel (2 pts), PCR amplification (1 pt)	Mean: D8.5 (D1-D24)	Bil TC with desaturation and unresponsiveness; multifocal TC and clon; bilat T; hypomotor followed by TC with alternating sides	Neonatal epilepsy: 4/4	4/4<br>Mean: 7.5 mo (range: 5-13)<br>Isolated seizures at 3.5, 6.5, 14.5y in 1	Mildly abn: 1<br>Ictal with contralateral EEG discharges: 1<br>NA: 2	-	Hypotonia: 1/4	Mild DD: 1<br>NA: 3/4	NA	NA	EA (Dizziness/unsteady gait possibly with associated with slurred speech, myoclonic jerks, distress, headache, pain, hypermotor activity, hyperventilation, retching, vomiting)	Mean: 24.3mo (range: 15mo-3.7y)	Poor drug response (Acetazolamide, 4-aminopyridine	NA	Mean: 11y (Range: 5-17y)	36
SCN2A	DEE11, OMIM #613721<br>BFIS3, #607745<br>EA9, #618924	R OA	4	4/9 NE + MD	AD	c.2960G > T p.S987I<br>c.4952T > G, p.F1651C<br>c.788C > T, p.A263V (x2)	missense<br>(presumed GoF variants)	Not performed	LP<br>LP<br>P	c.788C > T: 1 paternally inherited<br>1: mother tested negative, father unavailable<br>c.2960G > T; c.4952T > G: de novo	NA	Whole cohort: first 3 mo of life in 67%<br>D1-D10	Hemiclonic + apneas: 1<br>T-C: 3	BFIS3	NA	NA	NA	Hypotonia: 2	Mild GDD: 1<br>GDD (NA): 1<br>Average IQ; verbal and working, low average visual motor fluency, very low visual reasoning: 1<br>N: 1	-	-	EA: 4	Mean: 2.7y (range: 10mo-4.5y)	Ongoing: 4/4	NA	Mean: 9y (range: 2-16y)	37
SCN2A	DEE11, OMIM #613721<br>BFIS3, #607745<br>EA9, #618924	CR**?**	4	1	AD	c.788C > T, p.(Ala263Val)	missense	Not performed	P	De novo	NA	D2	T	BFIS3	NA	NA	NA	-	N	-	-	EA	20mo	ongoing	NA	5y	38

SCN2A	DEE11, OMIM #613721<br>BFIS3, #607745<br>EA9, #618924	CR	4	1	AD	c.788C>T, p.(Ala263Val)	missense	Not performed	P	De novo	NA	D1	T → GTC (often prolonged ; in clusters)	BFIS3	Seizure free: 7 mo	F-T sharp waves	N (4mo)	-	N	-	-	EA	15mo	ongoing	NA	19mo	39
SCN8A	EIEE13, OMIM #614558	R	4	Infantile DE/EE<br>22 (11F)<br>NE: 2/22<br>NE + MD: 1	AD	c.4639T>G, p.Phe1547Val	missense	Not performed (in silico prediction models used)	LP	De novo	NA	4m (median)<br>range: 0.5–36m	F<br>T<br>ES<br>TCS	DEE	Ongoing (reduced after 7y)	NA	Background deterioration: 22/22<br>FD (> T-P-O)	hypotonia	Severe ID, no SP/EC	Whole cohort: Cortical blindness: 17/22 Microcephaly/decelerating head growth: 4/22 (18%)	yes	Dyskinesia	NA	NA	Progressive cortical and subcortical atrophy posttrigonal WM T2 hyperintensity restricted diffusion in the optic radiations	22y	6
SCN8A	EIEE13, OMIM #614558	CR	4	1M	AD	c.3979A>G p.Ile1327Val	missense	Not performed (in silico prediction models used)	LP	De novo	WES + Sanger sequencing	birth	(NSE) tonic with apnea and bradycardia / focal tonic with eye squeezing and mouthing in clusters	EIEE	-	-	low-voltage background, interictal epileptiform T discharges; B-S-like in sleep	Dysmorphic features, congenital multiple arthrogryposis, hip dysplasia, inguinal hernia, hydrocele, kidney stones	Severe DD	-	-	coarse tremor, myoclonias<br>central hypotonia alternating with stiffness	At birth	-	N at birth, 5mo: mild F-T atrophy, CC thinning, 11mo: bilateral F-T atrophy, CC thinning, mild myelination delay	17 mo (died)	27
SCN8A	EIEE13, OMIM #614558	CR	4	1 M	AD	c.3979A>G p.Ile1327Val	missense	Not performed (previously described)	LP	De novo	NGS panel	birth	F clonic<br>Tonic + apnoea<br>NSE	EIEE	-	-	N background, C-P szs	PEG-fed<br>Central hypotonia + appendicular hypertonia<br>Acquired microcephaly	Severe DD	-	-	Exaggerated startle response paroxysmal posturing, jittery movements	birth	-	D7: N<br>mild cerebellar volume loss, age-appropriate myelination	NA	28
SCN8A	EIEE13, OMIM #614558	R OA	4	638 EOEE pts<br>19 pts with SCN8A<br>E + MD: 2<br>NE + MD: 1	AD	c.1763A>G	Missense?	Not performed	LP	De novo	Sanger sequencing of KCNQ2, STXBP1, SCN8A<br>Targeted NGS panel (64; 128 genes)	D1	T	DEE/EE	-	-	MFD: 1	Acquired microcephaly<br>PEG	Severe/profound	-	Regression: 1 (age NA)	Myoclonus: 1	Neonatal	NA	N	n=1 NA	25
SCN8A	EIEE13, OMIM #614558	R	4	17<br>E + MD: 12/17 (70.6%)<br>NE + MD: 2 (1F)	AD	c.4435A>G, p.Ile1479Val<br>c.5615G>T, p.Arg1872Leu	missense	Not performed	P: 2/2	De novo	NA	Range : 0-48 mo (Mean : 8.6 mo)<br>D1 : 1<br>1w : 1	GTCS: 2<br>FIAM: 1<br>Clon: 1<br>GT: 1<br>At Ab: 1<br>Multiple: 2/2	DEE: 2/2	-	N: 1<br>Slow: 1<br>FD + GD: 1	NA: 1<br>Ictal: 1	Hypotonia: 2<br>Microcephaly: 1<br>Torticollis: 1<br>Scoliosis: 1	Absent language: 1<br>Language delay: 1<br>Non-ambulatory: 1	-	yes	Ataxia: 1<br>Dystonia: 1	NA	NA	N: 1<br>Progressive cerebellar atrophy: 1<br>Progressive cerebral atrophy: 1	2y; 19y	26
SLC13A5	EIEE25, OMIM #615905	OA	4	8 pts from 4 families<br>NE + MD: 4/8	AR	Compound heterozygous: c.1022G>A, p.Trp341* c.1217_1217 dup11, p.Pro407Argfs *12<br>c.425C>T, p.Thr142Met c.655G>A, p.Gly219Arg<br>c.680C>T, p.Thr227Met c.1570G>C, p.Asp524His<br>Homozygous: c.1280C4T	Nonsense Frameshift<br>Missense Missense	Performed	P	Parents heterozygous carriers	WES	D1 : 4/4	F (clon): 4/4<br>SE: 4/4 (convulsive, hemiconvulsive, non-convulsive)<br>Fever-	EOEE	Seizure-free: 1 (3y)<br>Response to KD	MFD/shifting F:<br>(> post)<br>N: 1	MFD	Teeth hypoplasia/hypodontia: 100%<br>Microcephaly (none with NE + MD)<br>spasticity	DD (svere: ¾; mild-to-moderate: 1)	-	-	Ataxia: 2<br>choreathethosis: 2<br>athethosis: 1<br>dystonia: 1	NA	NA	N: 4/4 with NE + MD<br>periventricular leukomalacia-like abnormalities (none with NE + MD)	7.5y (4-10y)	30

						p.Ser427Leu	Missense missense<br><br>missense						sensitivity : 6/8														
SLC13A 5	EIEE25, OMIM #615905	OA	4	7 pts from 2 families<br><br>NE + MD: 3/7	AR	<u>Compound heterozygosis:</u> c.680C>T p.Thr227Met c.655G>A p.Gly219Arg<br><br>c.680C>T p.Thr227Met c.655G>A p.Gly219Arg<br><br><u>Homozygosis</u>: c.1463T>C p.Leu488Pro	missense	Not performed (in silico prediction models used)	LP: 3/3	Parents heterozygou s carriers	WES	2h-1st week	SE: 6/7<br><br>Subtle: 5/7<br><br>Clon/hemi clonic: 2/7<br><br>NA: 1/7<br><br>Fever-sensitivity	EOEE	2/7 (age: NA)	T-O dischar ges (shifting hemisp heres): 1<br><br>FD (T): 2<br><br>MFD: 1<br><br>Low voltage backgro und: 1<br><br>NA: 4	NA	Widely spaced teeth, no facial dismorphisms/ hypodontia<br><br>Axial hypotonia: 6/7<br><br>Peripheral hypertonia: 5/7	Profound: 5/7<br><br>Several words: 2/2<br><br>Walking: 1/7	-	-	Ataxia: 1/7<br><br>Choreo-athethos is: 2/7<br><br>Chorea: 1/7<br><br>Dystonia : 2/7<br><br>Dyskine sia: 1/7<br><br>No MD: 3/7	NA	NA	"no signs of perinatal insult"	Mean: 6.7 y (range: 3-14y)	29
SMC1A	EIEE85, OMIM #301044	Single case	4	1F	XLD	c.1911 + 1G>T p. Thr638Valfs*48	frameshift	Performed	P	De novo	WES	During the first month of life	Eyelid myoclonia	DEE	ongoing	F	Hyps	IUGR, Primary microcephaly<br><br>dysmorphisms<br><br>spastic tetraparesis<br><br>scoliosis<br><br>GERD	Severe ID (non-verbal)	Poor eye contact	NA	Hand stereoty pies	2y	NA	small frontal lobes, thin CC	7y	42
SYNJ1	EIEE53 OMIM #617389<br><br>PARK20 OMIM #615530	OA	4	6 E + MD<br><br>(NE: 2/6)<br><br>NE + MD: 2/6	AR	c.1938delT c.3365-2A4G	Premature stop	performed	P	Inherited from heterozygou s parents	WES	D1, D12	eye blinking + "shoulder movemen ts" :1<br><br>jerking/ey e deviation: 1<br><br>→ TC, myo: 2/2	DEE	refractory	MFD: 2/2 slow backgro und: 1<br><br>Severel y abnorm al: 1	Severely abnormal (discontinu ous): 1<br><br>slow backgroun d + MFD: 1	Hypotonia → hypertonia<br><br>Progressive spastic quadriparesis	Profound DD	Cortical visual impairment: 1	Progres sive neurolo gical decline	Dystonia opistoto nus: 2/6	infancy	-	N (MRS with large creatine peak): 1<br><br>thin CC, periventricula r WM gliosis and atrophy: 1	Died: 2.5y<br><br>Died: 8y	40
SYNJ1	EIEE53 OMIM #617389<br><br>PARK20 OMIM #615530	CR	4	1 (F)	AR	Biallelic c.858_862delACAAA	frameshift	Not performed	P	NA	WES	D2	Eye twitching, hypertonu s and bicycling movemen ts → WS (8mo), myo	EOEE	refractory	NA	NA	Profound hypotonia → severe spastic quadriparesis	profound	Severe cortical visual impairment	-	Opistoto nic posturin g	infancy	Part ial res pon se to clon aze pa m	N	28mo	41
STXBP1	EIEE4, OMIM #612164	R	4	CR 1 F	AD	c.416C>T	Missense	Not performed (in silico prediction models used)	P/LP	De novo	WES	D5	T + focal	DEE	-	-	MFD on N backgroun d	Respiratory chain complex I profound deficit	GDD	Severe language and cognitive impairment	yes	Marked ataxia<br><br>Head nodding<br><br>Dystonic posturin g + tremor<br><br>resting tremor, cogwhee l rigidity and hypomi mia +	6y<br><br>7y<br><br>9y<br><br>12y	-	N (4mo; 6y)	12y	34

																						pyramidal signs					
STXBP1	EIEE4, OMIM #612164	R	4	Case series<br>5 (1F)<br>NE + MD: 2/5	AD	c.1702 +1G>A /<br>c.875G>A p.Arg292His	Splice site<br>missense	Not performed	P: 2/2	De novo	NA	Range : D1-8y (mean: 22.6m)	Onset My: 1/2 T: 1/2 CT: 1 Multiple types: 1<br>Follow-up:<br>FIA: 2<br>T ES Gelastic	DEE	-	Slow, asymmetrical IED<br>Bilat independent F ictal activity	MFD: 1<br>FD: 1	Dysmorphisms<br>LL's spasticity<br>polymyoclonus	Severe: 4/5 Moderate: 1/5	Bruxism: 4/5	-	Choreoathetosis<br>focal dystonia<br>startles<br>stereotypies<br>head tremor; trunk dyskinesia; UUL's resting and postural tremor, cogwheel rigidity	NA	NA	Atrophy	33y ; 45y	33
**STXBP1**	EIEE4, OMIM #612164	OA	4	5 positive from a cohort of 52	AD	c.902+lG>A p.Q301fsX1<br>c.1720A>C p.T574P<br>del exon 8-14<br>c.548T>G p.L183R	Frameshift<br>Missense<br>Intragenic deletion<br>missense	Not performed	P<br>del exon 8-14 : LP	De novo	Sequencing + array-CGH	Neonatal 4/5	Onset Clonic: 2 ES: 2 Follow-up ES: 3 F: 1 TC: 1	DEE	4/4<br>6mo: 1<br>12mo: 1<br>18mo: 1	B-S (more continuous after 2 mo)	-	Hypotonia: 3<br>FFT: 1<br>Startles: 2	GDD: 4<br>Nonverbal: 4<br>Non-ambulant: 1	Autistic features: 3	-	myoclonus: 2<br>tremor: 2<br>choreic: 4<br>Dyskinetic: 2<br>Stereotypies: 2<br>Ataxia: 2	Since birth<br>> 2y<br>4y	-	No cortical malformations	mean: 4.25 y, range: 2y-7y	31
STXBP1	EIEE4, OMIM #612164		4	137<br>Pos: 13<br>E + MD: 6 (3M)<br>NE + MD: 1 (STXBP1)	AD	c.296A>G p.Tyr99Cys	missense	Not performed (in silico prediction models used)	LP	De novo	WES	Neonatal	T<br>F	EOEE<br>non-specific multifocal	seizure-free, 6y	MFD	-	Hypotonia	Profound ID	Autistic features	-	Stereotypies	NA	NA	N	7y	32
VAMP2	NDD with hypotonia and autistic features with or without hyperkinetic movements, OMIM #618760	R OA	4	5 unrelated pts E + MD: 2/5 (2 M)<br>NE + MD: 1/2	AD	c.233A>C p.Glu78Ala	missense	Performed	P	De novo	WES	1 at birth	Focal<br>GTCS	-	-	Ictal: fast rhythmic activity followed by sharp-and-slow-wave complexes	-	Hypotonia<br>CVI	Severe ID Non verbal<br>non ambulant	ASD<br>Rett-like features<br>self-injury	-	Chorea	NA	NA	unremarkable	10y	48

WWOX	EIEE28,<br>OMIM<br>#616211	R<br>OA	4	E + MD: 18/20<br>NE + MD: 5/20<br>NE + possible MD: 1	AR	Compound heterozygous: c.173-1G>T c.517_1056dup p.[Asp58Alafs*3];[His173_Met352dup]<br>c.231_409del c.606_791del p.[Asp77Glufs*27];[Pro203_Arg264del]<br>c.953C>T c.517_1056del p.[Ser318Leu];[His173_Met352del]<br>c.410_516del c.606_791del p.[Gly137Alafs*2];[Pro203_Arg264del]<br>c.410G>A c.517_791del p.[Gly137Glu];[His173Ilefs*5]<br>c.517_1056del c.705dupG p.[His173_Met352del];[His236Alafs*34]	Missense: 2<br>Frameshift: 5<br>Intragenic deletions: 4<br>Intragenic duplications: 1	Not performed (in silico prediction models used)	LP: c.173-1G>T, c.953C>T, c.410G>A<br>P: c.517_1056dup, c.231_409del c.606_791del, c.517_1056del, c.410_516del c.606_791del, c.517_791del c.517_1056del c.705dupG	Parents heterozygous carriers	Array-CG/WES/WGS	Whole cohort: Range: D1-7mo mean: 1.7 mo (n=1 NA)<br>NE: median D2, mean D7 (range: D1-D20)	Onset: F clonic: Subtle: 1 F: 2 (1 occipital) ES: 2<br>F/U: Ab: 1 T-C: 2 T: 1 ES: 2	EOEE (“WOREE”)	-	Slow: 2<br>Abn: 1<br>disorganized: 2<br>Hyps: 1<br>Mod Hyps: 1<br>FD: 3 (> post)<br>MFD: 1	Disorganized without IED: 1/5<br>NA: 4/5	Hypotonia: 4<br>pyramidal signs/hypertonia: 100%<br>early-onset scoliosis/kiphosis: 100%<br>Poor/absent eye contact: 4<br>Abnormal ERG: 1<br>Abnormal VEP: 1	Profound DD	-	-	Hypokinesia: 5<br>pedalling and boxing: 1<br>dystonic: UULL's 2<br>Myoclonus: 1<br>Startle: 1<br>Paroxysmal involuntary movements: 1	NA	-	N: 1<br>thin CC: 2<br>hypoplastic CC: 2<br>increased CSF spaces: 2<br>abn lateral ventricles: 1	16.7mo (range: 3mo-3y5mo)	40

Table legend. List of abbreviations: AA: amino acids, Ab: absence, abn: abnormal, At: atonic, CC: corpus callosum, CI: cognitive impairment, CP: cerebral palsy, CR: case report, CSF: cerebrospinal fluid, CVI: central visual impairment, DD: developmental delay, DE: developmental encephalopathy, E: epilepsy, EE: epileptic encephalopathy, EEG: electroencephalogram, ENC: encephalopathy, EPC: Epilepsia partialis continua, ES: epileptic spasms, ESES: electrical status during slow waves sleep, F: female, FIAS: focal impaired awareness seizures, FD: focal discharges, FSE: febrile status epilepticus; FTT: failure to thrive, F/U: follow-up, FS: febrile seizure, GD: generalized discharges, GDD: global developmental delay, Gel: gelastic, GERD: gatro-esophageal reflux disease, HC: hemiclonic, HD: head drop, HyCN: hypoplasia of the caudate nuceus, Hyps: hypsarrhythmia, IS: infantile spasms, L: language, M: male, MFD: multifocal discharges, m: month(s), MD: movement disorder, MIPS: Molecular Inversion Probe Sequencing, MRI: magnetic resonance imaging, Mt: motor, My: myoclonic, MyAb: myoclonic absence, N: novel, n: number, NA: not available, NAA: N-acetyl-aspartate, NGS: next-generation sequencing, Ny: nystagmus, OA: organic acids, OMA: oculo-motor apraxia, OR: optic radiations, PCH: pontocerebellar hypoplasia, PKD: paroxysmal kinesigenic dyskinesia, PPR: photoparoxysmal response, pt(s): patient(s), Pu: published, PV: pathogenic variants, PSW: poly-spikes-and-waves, R: retrospective, Ref: reference, S: suspended, SE: status epilepticus, SGT: single gene testing, SW: spike-and-wave complexes, T: tonic, TC: tonic-clonic, VE: ventricular enlargement, VI: visual impairment, VM: ventriculomegaly; y: year(s), w: weeks, WES: whole exome sequencing,
